# Preoperative-to-Postoperative Radiological Outcomes Following Open Reduction and Internal Fixation With Volar Locking Plate in Adult Distal Radius Fractures: A Prospective Observational Study

**DOI:** 10.7759/cureus.108644

**Published:** 2026-05-11

**Authors:** Aditya K Singh, Indrajit D Bhoumik, Abhishek Choudhary

**Affiliations:** 1 Department of Orthopedics, Government Doon Medical College, Dehradun, IND

**Keywords:** distal radius fracture, orif, radiological outcome, sarmiento criteria, volar locking plate

## Abstract

Background and aim

Distal radius fractures (DFRs) are among the most common orthopedic injuries, particularly in elderly and postmenopausal women due to osteoporosis and low-energy trauma, such as falls on an outstretched hand. Restoration of anatomical alignment is essential for optimal functional outcomes, as residual deformity can lead to stiffness, reduced grip strength, malunion, and early degenerative changes. With evolving treatment strategies, open reduction and internal fixation (ORIF) using volar locking plates has become a preferred modality due to its biomechanical stability and facilitation of early mobilization. This study aimed to evaluate postoperative radiological outcomes following volar locking plate fixation and compare them with preoperative parameters.

Methods

This prospective observational study was conducted over a period of 10 months at a tertiary care center. A total of 50 adult patients with radiographically confirmed closed DFRs were included based on predefined inclusion and exclusion criteria. All patients underwent ORIF using a volar locking plate through a standard volar approach. Preoperative and postoperative radiographs were obtained in AP and lateral views. Radiological parameters assessed included dorsal tilt (expressed postoperatively as loss of palmar tilt), radial inclination, radial height, ulnar variance, and loss of radial deviation. A paired t-test and chi-square test were used for statistical analysis, and outcomes were evaluated using Sarmiento’s modification of Lindstrom’s criteria.

Results

The mean age of the study population was 50 ± 13 years, with a predominance of female patients (60%). The most common mechanism of injury was fall on an outstretched hand, observed in 76% of cases. Significant improvement was observed in all radiological parameters following surgical intervention. The mean dorsal tilt improved from 12 ± 4° preoperatively to a postoperative loss of palmar tilt of 0.38 ± 2°. Radial inclination increased from 14.8 ± 2° to 19.8 ± 2°, while radial height improved from 7.98 ± 1 mm to 10.98 ± 1 mm. Ulnar variance decreased from 5 ± 2 mm to 2 ± 2 mm. The mean postoperative loss of radial deviation was 2.8 ± 2°. All observed changes were statistically significant (p < 0.001). Distribution-based analysis demonstrated a marked shift from abnormal preoperative ranges to near-normal postoperative values across all parameters. According to Sarmiento’s grading system, 35 (70%) patients achieved excellent outcomes and 15 (30%) achieved good outcomes, with no cases classified as fair or poor. Residual deformity was insignificant in 70% of patients and slight in 30%, with no moderate or severe deformities observed.

Conclusions

ORIF using a volar locking plate demonstrated favorable immediate postoperative radiological restoration in DFRs and may be considered a reliable option for achieving anatomical alignment. Most patients achieved satisfactory radiological correction with minimal residual deformity in the immediate postoperative period. However, as the present study focused specifically on early radiological outcomes following fixation, further studies incorporating long-term follow-up, functional assessment, fracture union, and complication analysis are required to determine overall clinical effectiveness and long-term outcomes.

## Introduction

Distal radius fractures (DRFs) are among the most commonly encountered fractures in orthopedic practice, accounting for nearly 20% of fractures treated in emergency care [1]. First described by Sir Abraham Colles in 1814, these fractures are particularly prevalent in women, largely attributed to osteoporosis, where even low-energy trauma such as minor falls can result in fracture. The most frequent mechanism of injury is a fall on an outstretched hand (FOOSH), with the fracture pattern influenced by the magnitude of force, wrist position at the time of impact, and underlying bone quality [2].

Over time, multiple treatment strategies have been explored for DRF. Traditional conservative management, including closed reduction and casting under anesthesia, has often been associated with suboptimal functional outcomes, residual deformity, and long-term disability [3]. Limitations of conservative methods have led to the evolution of minimally invasive and operative techniques aimed at improving anatomical restoration and functional recovery. Techniques such as percutaneous K-wire fixation and external fixation offer some advantages; however, achieving precise anatomical reduction, particularly in intra-articular and unstable fractures, remains challenging with these approaches [4].

Operative management, especially open reduction and internal fixation (ORIF), has gained prominence as it allows direct visualization, accurate fracture reduction, and stable fixation. Among the various fixation methods, volar locking plate fixation (LPF) has emerged as a preferred modality due to its biomechanical stability, facilitation of early mobilization, and improved functional and radiological outcomes. This technique is particularly advantageous in osteoporotic bone, where conventional fixation methods may fail to provide adequate stability [5].

The primary goals of osteosynthesis in DRF include restoration of the articular surface, anatomical alignment of fracture fragments, and reestablishment of key radiological parameters such as radial height, radial inclination, palmar tilt, and ulnar variance [6]. These parameters are critical determinants of wrist biomechanics and functional outcomes. Inadequate restoration can lead to complications such as malunion, decreased grip strength, restricted range of motion, and early onset osteoarthritis [7]. Previous studies have demonstrated that even minor deviations in these radiological indices can significantly impact clinical outcomes. For instance, malunion has been associated with wrist deformity and stiffness, while residual deformity correlates with reduced grip strength [8,9]. Additionally, axial shortening of the radius, even without articular incongruity, has been shown to increase the risk of permanent disability [10].

Radiological evaluation plays a crucial role in both preoperative planning and postoperative assessment of DRF. Standard parameters such as palmar tilt (lateral view), radial length and inclination (AP view), and ulnar variance are routinely used to assess fracture reduction and alignment. Acceptable radiological criteria have been defined to guide treatment adequacy and predict outcomes. Furthermore, scoring systems such as Sarmiento’s modification of Lindstrom criteria provide a structured approach to evaluate overall radiological results [11].

Despite advancements in surgical techniques and fixation devices, variability still exists in achieving optimal radiological outcomes. Factors such as fracture pattern, bone quality, surgical technique, and implant positioning can influence postoperative alignment and stability. Therefore, systematic evaluation of radiological parameters following ORIF with volar plating is essential to assess the effectiveness of this widely adopted technique.

In this context, the present hospital-based observational study is designed to evaluate the postoperative radiological outcomes of DFRs treated with ORIF using volar plating. The primary objective of this study was to evaluate immediate postoperative radiological restoration following ORIF with volar LPF in DFRs. The secondary objective was to assess overall radiological outcomes using Sarmiento’s modification of Lindstrom criteria.

## Materials and methods

Study design, setting, population, and duration

**Figure 1:**
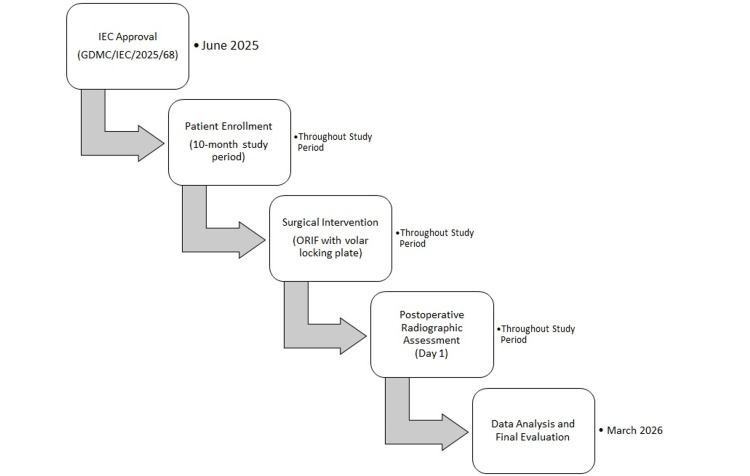
Timeline of the study protocol from institutional ethics approval (June 2025) through patient enrollment, surgical intervention, follow-up, and final data analysis over a 10-month period GDMC, Government Doon Medical College; ORIF, open reduction and internal fixation

**Figure 2:**
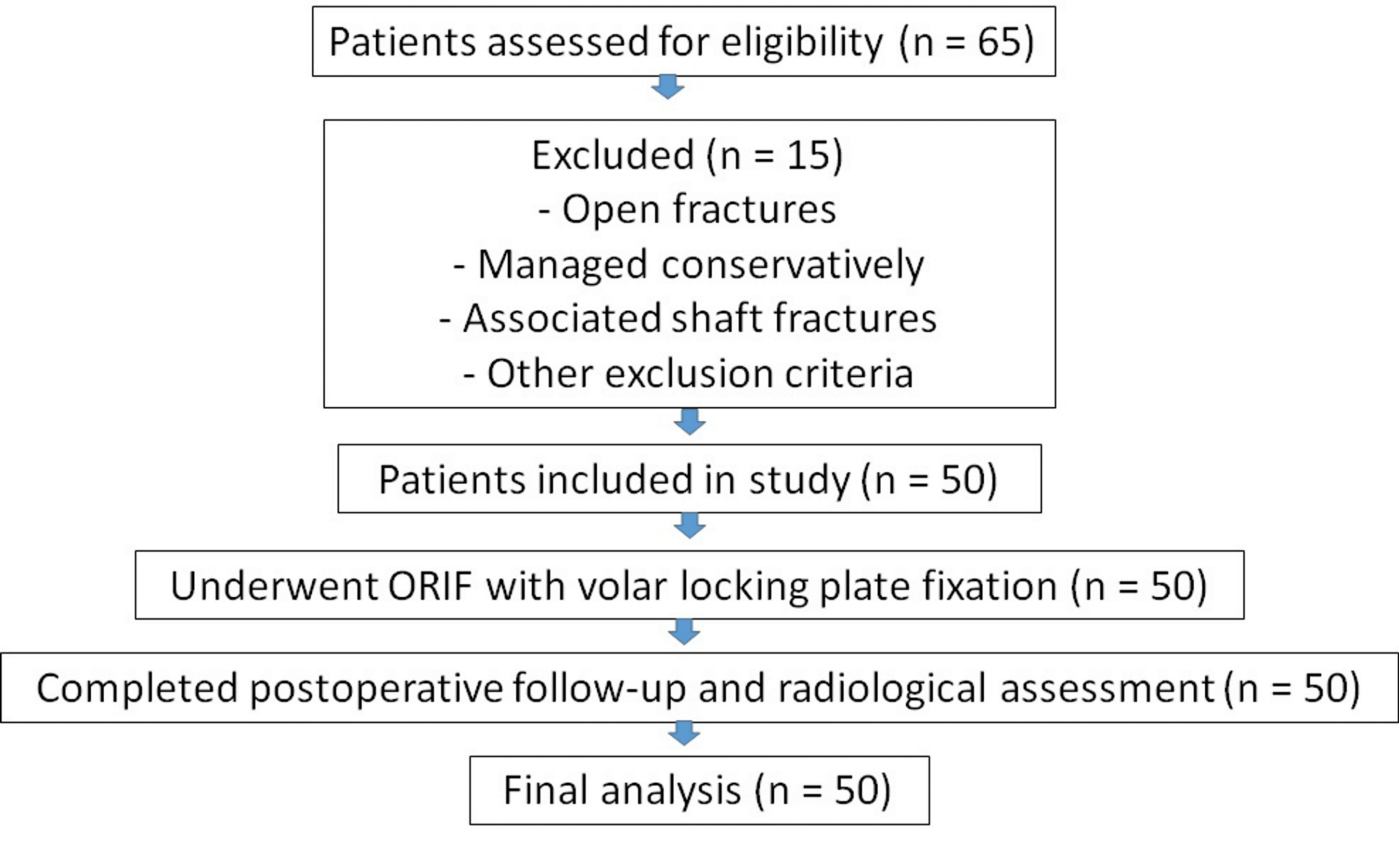
Flowchart depicting patient selection, exclusions, and final study cohort ORIF, open reduction and internal fixation

This prospective observational study was carried out over a period of 10 months (June 2025 to March 2026) following approval from the Institutional Ethics Committee (approval GDMC/IEC/2025/68) at Government Doon Medical College, Dehradun, in the Department of Orthopedics. Patients presenting to both the orthopedics outpatient department and emergency services were screened for eligibility. The study population comprised patients diagnosed with DRF confirmed radiographically, and only those meeting the predefined inclusion and exclusion criteria were enrolled after obtaining informed written consent (Figure 1). Participant selection is illustrated in Figure 2.

Sample size

The sample size was calculated using the following formula:

$n = \frac{(Z_{\alpha/2})^{2} \times (P \times Q)}{E^{2}},$

where Zα/2 = 1.96 at 5% error, P = 92% (assumed proportion of good to excellent outcomes), Q = 1 − P, and E = 10% allowable error. Based on this calculation, the final sample size was determined to be 50 patients.

Eligibility criteria

A total of 65 patients were screened, of which 50 were included and 15 were excluded based on predefined criteria. Patients aged 18 years and above with closed DFRs, including intra-articular, extra-articular, or comminuted fractures, treated with ORIF using a volar plate and available for postoperative radiographic follow-up were included (n = 50). Patients unfit for surgery, those with associated shaft fractures of the radius, open fractures, or those treated conservatively, with K-wire fixation, external fixation, or dorsal plating were excluded from the study (n = 15) (Figure 2).

Data collection and clinical evaluation

A detailed history was obtained for each patient, including the duration since injury and relevant medical history, followed by a thorough clinical examination. All patients underwent necessary preoperative investigations and radiographic assessment as per institutional protocol. Fractures were classified using AO and Frykman classification systems [12].

Radiological assessment

Preoperative and postoperative radiographs of the wrist in AP and lateral views were obtained to assess key parameters including dorsal tilt and postoperative loss of palmar tilt, radial height, radial inclination, and ulnar variance. Radiographic measurements were independently assessed by two orthopedic surgeons using standardized measurement techniques to minimize observer-related variability. Standard definitions were used for each parameter to ensure consistency in measurement. Radial height was measured as the distance between two parallel lines drawn perpendicular to the longitudinal axis of the radius, one at the tip of the radial styloid and the other at the level of the ulnar corner of the distal radius. Ulnar variance was measured as the relative length difference between the distal articular surfaces of the ulna and radius. Loss of radial deviation was determined by comparing postoperative radial inclination with accepted normal anatomical values.

**Figure 3:**
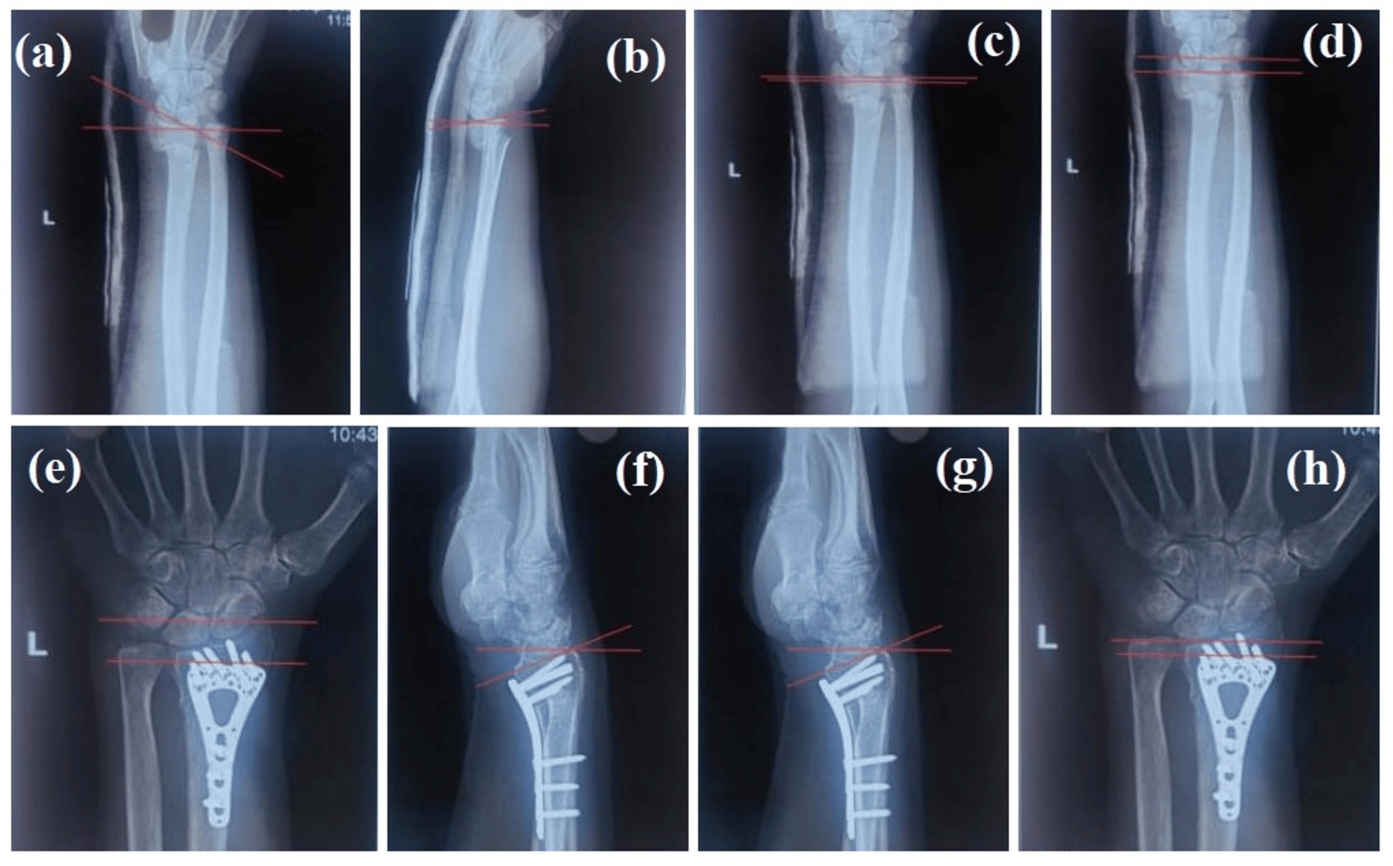
Representative preoperative and postoperative radiographs demonstrating measurement of radial height, radial inclination, dorsal tilt, and ulnar variance (a) Preoperative radial inclination = 14º; (b) Preoperative dorsal tilt = 13º; (c) Preoperative ulnar variance = 4 mm; (d) Preoperative radial height = 8 mm; (e) Postoperative radial height = 10 mm; (f) Postoperative loss of palmar tilt = 0º; (g) Postoperative radial inclination = 20º; (h) Postoperative ulnar variance = 2 mm. Measurement landmarks are indicated to illustrate radiological assessment parameters.

Figure 3 demonstrates the measurement landmarks for radial height, radial inclination, dorsal tilt, and ulnar variance. Although surgeries were performed by different orthopedic surgeons within the department, the operative protocol and implant system remained standardized across all cases.

Surgical procedure

**Figure 4:**
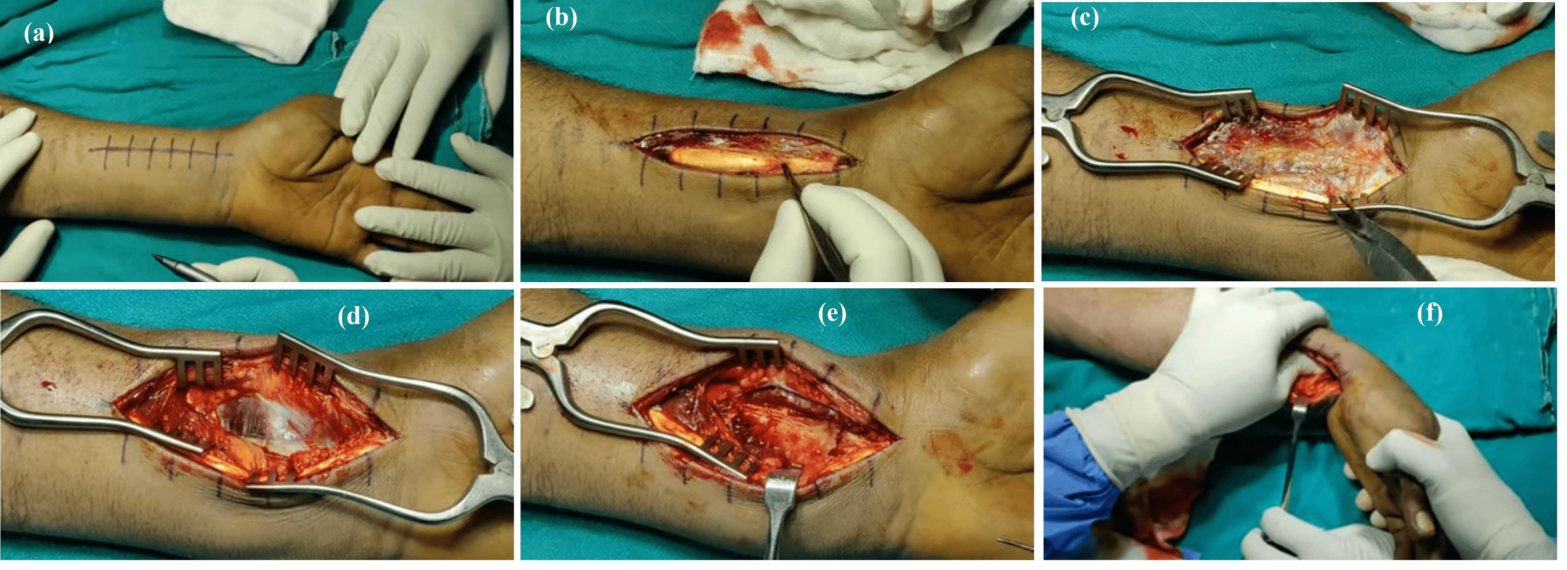
Steps of volar approach for DRF fixation: (a) skin marking for modified Henry’s approach; (b) longitudinal incision over volar aspect of distal forearm; (c) soft tissue dissection with retraction of surrounding structures; (d) exposure of pronator quadratus muscle; (e) elevation/incision of pronator quadratus to expose fracture site; (f) intraoperative reduction of fracture with traction DRF, distal radius fracture

**Figure 5:**
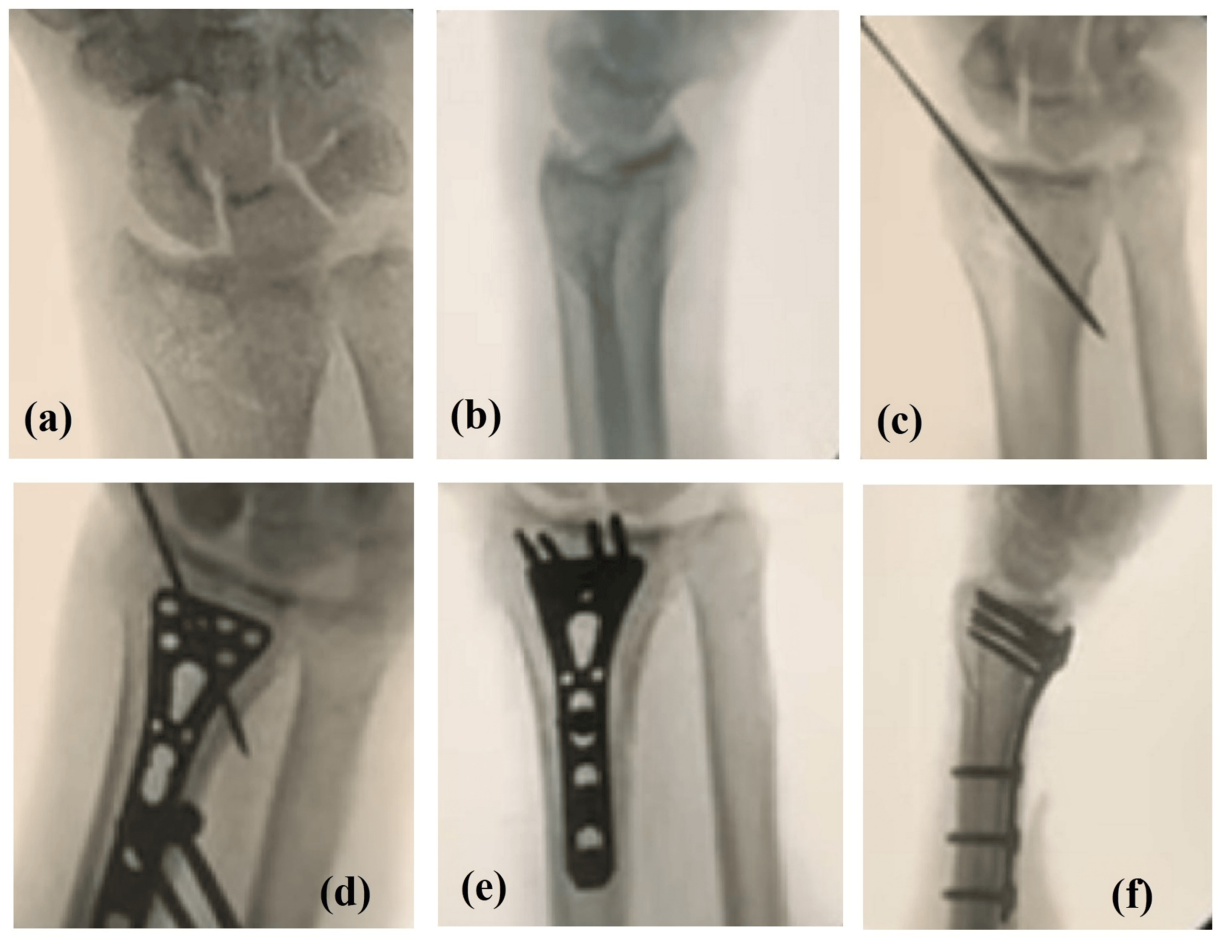
Radiographic sequence of DRF management (a) Preoperative AP view showing fracture; (b) preoperative lateral view; (c) intraoperative reduction with K-wire fixation; (d) intraoperative image showing placement of volar locking plate; (e) postoperative AP view demonstrating plate fixation; (f) postoperative lateral view showing restored alignment. DRF, distal radius fracture

All patients underwent ORIF using a standard volar (modified Henry’s) approach. The procedure was performed with the patient in the supine position under regional or general anesthesia with tourniquet control. After skin incision and soft tissue dissection, the pronator quadratus muscle was elevated to expose the distal radius. Fracture reduction was achieved under fluoroscopic guidance and temporarily stabilized using Kirschner wires. Definitive fixation was performed using a precontoured 2.4 mm volar locking compression plate. Proper plate positioning and screw placement were confirmed intraoperatively using C-arm imaging in both AP and lateral views. The pronator quadratus was repaired where feasible, and the wound was closed in layers after adequate irrigation (Figure 4, Figure 5).

Postoperative care

Postoperatively, patients received intravenous antibiotics and analgesics, followed by additional analgesics as required. Radiographs were obtained on the first postoperative day. Preoperative and postoperative radiological parameters were compared to assess the effectiveness of surgical intervention.

Outcome measures

Radiological outcomes were evaluated based on changes in palmar tilt, radial height, radial inclination, and ulnar variance. Final radiological outcomes were categorized using Sarmiento’s modification of Lindstrom’s criteria, which classifies results into excellent, good, fair, and poor based on predefined thresholds of residual deformity, including loss of palmar tilt, radial shortening, and loss of radial deviation. Each parameter was measured using standardized techniques on AP and lateral radiographs to ensure consistency.

Statistical analysis

Data were recorded in a predesigned proforma. Qualitative variables were expressed as frequencies and percentages, while quantitative variables were presented as mean ± SD. A paired t-test was used to compare preoperative and postoperative continuous variables. The chi-square test was applied for categorical variables in distribution-based analyses. A p-value < 0.05 was considered statistically significant. Statistical analysis was performed using IBM SPSS Statistics for Windows, version 26.0 (released 2018; IBM Corp., Armonk, NY, USA).

## Results

The mean age of the study population was 50 ± 13 years. The majority of patients belonged to the ≥61 years age group (17, 34%), followed by 51-60 years (11, 22%) and 41-50 years (9, 18%), while comparatively fewer patients were observed in the younger age groups. Females constituted the majority of the study population (30, 60%), whereas males accounted for 20 (40%). The left side was slightly more commonly affected (26, 52%) than the right side (24, 48%). The most common mode of injury was FOOSH, observed in 38 (76%) patients, while road traffic accidents accounted for 12 (24%) cases (Table 1).

**Table 1:** Demographic and baseline characteristics FOOSH, fall on an outstretched hand

Variable	Domain	N (%)
Age group (years)	≤20	1 (2%)
	21-30	5 (10%)
	31-40	7 (14%)
	41-50	9 (18%)
	51-60	11 (22%)
	≥61	17 (34%)
Gender	Female	30 (60%)
	Male	20 (40%)
Side affected	Left	26 (52%)
	Right	24 (48%)
Mode of injury	Road traffic accidents	12 (24%)
	FOOSH	38 (76%)

The mean preoperative dorsal tilt was 12 ± 4°, which improved to a postoperative loss of palmar tilt of 0.38 ± 2°, with a mean difference of 11.62° (t = 23.25, 95% CI: 10.60-12.60, p < 0.001). The mean radial inclination increased from 14.8 ± 2° preoperatively to 19.8 ± 2° postoperatively, with a mean difference of 5° (t = 19.77, 95% CI: 4.22-5.18, p < 0.001). The mean radial height improved from 7.98 ± 1 mm to 10.98 ± 1 mm postoperatively, with a mean difference of 3 mm (t = 25.58, 95% CI: 3.17-3.71, p < 0.001). The mean ulnar variance decreased from 5 ± 2 mm preoperatively to 2 ± 2 mm postoperatively, with a mean difference of 3 mm (t = 13.27, 95% CI: 2.5-3.5, p < 0.001). The mean postoperative loss of radial deviation was 2.8 ± 2° (Table 2).

**Table 2:** Radiological parameters (pre- and postoperative) Data expressed as mean ± SD. Paired t-test applied.

Parameter	Pre-op (mean ± SD)	Post-op (mean ± SD)	Mean difference	t-value	95% CI	p-value
Loss of palmar tilt (°)	12 ± 4	0.38 ± 2	11.62	23.25	10.60-12.60	<0.001
Radial inclination (°)	14.8 ± 2	19.8 ± 2	5	19.77	4.22-5.18	<0.001
Radial height (mm)	7.98 ± 1	10.98 ± 1	3	25.58	3.17-3.71	<0.001
Ulnar variance (mm)	5 ± 2	2 ± 2	3	13.27	2.5-3.5	<0.001
Loss of radial deviation (°)	-	2.8 ± 2	-	-	-	-

Preoperatively, the majority of patients had a dorsal tilt in the range of 10-14° (22, 44%), followed by 5-9° (15, 30%) and >14° (13, 26%), with no patients having a 0° tilt. Postoperatively, 43 (86%) patients achieved a 0° loss of palmar tilt, while seven (14%) had a residual loss of palmar tilt in the range of 1-4°, and no patient had a loss of palmar tilt greater than 4°. This change was statistically significant (χ² = 100.0, p < 0.0001) (Table 3).

**Table 3:** Dorsal tilt and loss of palmar tilt distribution (pre- vs postoperative) Chi-square test applied.

Range (°)	Pre-op, N (%)	Post-op, N (%)	Statistics
0	0 (0%)	43 (86%)	χ² = 100.0, p < 0.0001
1-4	0 (0%)	7 (14%)	
5-9	15 (30%)	0 (0%)	
10-14	22 (44%)	0 (0%)	
>14	13 (26%)	0 (0%)	

Preoperatively, the majority of patients had radial height in the range of 6-10 mm (48, 96%), while two (4%) patients were in the 1-5 mm range, and none were in the 11-12 mm range. Postoperatively, 32 (64%) patients achieved a radial height of 11-12 mm, while 18 (36%) were in the 6-10 mm range, and no patients remained in the 1-5 mm category. This improvement was statistically significant (χ² = 47.64, p < 0.0001) (Table 4).

**Table 4:** Radial height distribution (pre- vs postoperative) Chi-square test applied.

Range (mm)	Pre-op, N (%)	Post-op, N (%)	Statistics
1-5	2 (4%)	0 (0%)	χ² = 47.64, p < 0.0001
6-10	48 (96%)	18 (36%)	
11-12	0 (0%)	32 (64%)	

Preoperatively, the majority of patients had radial inclination in the range of 10-15° (38, 76%), followed by 16-20° (12, 24%), with none in the 21-22° range. Postoperatively, the majority of patients shifted to the 16-20° range (42, 84%), while eight (16%) patients achieved 21-22°, and none remained in the 10-15° range. This improvement was statistically significant (χ² = 62.67, p < 0.0001) (Table 5).

**Table 5:** Radial inclination distribution (pre- vs postoperative) Chi-square test applied.

Range (°)	Pre-op, N (%)	Post-op, N (%)	Statistics
10-15	38 (76%)	0 (0%)	χ² = 62.67, p < 0.0001
16-20	12 (24%)	42 (84%)	
21-22	0 (0%)	8 (16%)	

Preoperatively, the majority of patients had ulnar variance in the range of 1-4 mm (25, 50%), followed by 5-7 mm (16, 32%), 8-11 mm (6, 12%), and >11 mm (1, 2%), while only 2 (4%) patients had ulnar variance <1 mm. Postoperatively, the majority of patients had ulnar variance <1 mm (38, 76%), followed by 1-4 mm (11, 22%), and 5-7 mm (1, 2%), with no patients in higher ranges. This change was statistically significant (χ² = 100.0, p < 0.0001) (Table 6).

**Table 6:** Ulnar variance distribution (pre- vs postoperative) Chi-square test applied.

Range (mm)	Pre-op, N (%)	Post-op, N (%)	Statistics
<1	2 (4%)	38 (76%)	χ² = 100.0, p < 0.0001
1-4	25 (50%)	11 (22%)	
5-7	16 (32%)	1 (2%)	
8-11	6 (12%)	0 (0%)	
>11	1 (2%)	0 (0%)	

The majority of patients had excellent radiological outcomes, observed in 35 (70%) cases, while 15 (30%) had good outcomes. No patients were classified as fair or poor. Regarding residual deformity, 35 (70%) patients had insignificant deformity, while 15 (30%) had slight deformity, and none had moderate or severe deformity (Table 7).

**Table 7:** Radiological outcome and residual deformity

Variable	Domain	N (%)
Sarmiento grade	Excellent	35 (70%)
	Good	15 (30%)
	Fair	0 (0%)
	Poor	0 (0%)
Residual deformity	Insignificant	35 (70%)
	Slight	15 (30%)
	Moderate	0 (0%)
	Severe	0 (0%)

## Discussion

The primary goal in the management of DRFs is to achieve anatomical reduction and maintain stability to restore optimal wrist function. Inadequate reduction may result in malunion, leading to poor functional outcomes such as stiffness, decreased grip strength, and post-traumatic arthritis. Fracture stability plays a critical role in maintaining reduction, particularly in unstable fracture patterns where there is a higher risk of secondary displacement and collapse. Restoration of anatomical alignment is therefore essential not only for radiological correction but also for preserving long-term wrist biomechanics and function. The present study evaluated postoperative radiological outcomes of DRF treated with ORIF using volar plating, with assessment based on Sarmiento’s modification of Lindstrom’s criteria.

The mean age in the present study was 50 ± 13 years, with the majority of patients aged ≥50 years, indicating a higher incidence in older individuals. This finding is comparable to studies by Hanumanthappa et al. [13] and Kotian et al. [14], which also reported higher mean ages, suggesting increased susceptibility in the elderly population. A female predominance (60%) was observed, consistent with Hanumanthappa et al. [13], although other studies such as Kotian et al. [14] and Sakhuja et al. [15] reported male predominance. The higher proportion of females in the present study may be attributed to osteoporosis, predisposing to fractures following low-energy trauma. Age-related bone fragility and decreased cortical thickness further contribute to fracture severity and may influence the choice of fixation method, making volar locking plates particularly advantageous in osteoporotic bone. The left side was slightly more commonly affected (52%), similar to findings by Hanumanthappa et al. [13] and Khan et al. [16]. The most common mechanism of injury was FOOSH in 76% of cases, which is consistent with previous studies reporting falls as the predominant cause of DRF, especially in older individuals [13,14].

In the present study, significant improvement was observed in all radiological parameters following surgical intervention. The mean radial height improved from 7.98 ± 1 mm preoperatively to 10.98 ± 1 mm postoperatively, indicating effective restoration of radial length and correction of impaction. Restoration of radial height is crucial, as even minimal shortening can alter load transmission across the wrist joint and increase stress on the ulnocarpal articulation. Radial inclination increased from 14.8 ± 2° preoperatively to 19.8 ± 2° postoperatively, with a mean improvement of 5°, which is comparable to results reported by Hanumanthappa et al. [13] and Khan et al. [16]. Proper restoration of radial inclination contributes to maintaining normal wrist kinematics and distribution of forces across the radiocarpal joint. The mean postoperative loss of palmar tilt was 0.38 ± 2°, with 86% of patients achieving neutral tilt, indicating near-anatomical restoration. Maintenance of palmar tilt is particularly important, as dorsal angulation has been associated with decreased wrist flexion and altered carpal mechanics. Ulnar variance decreased from 5 ± 2 mm preoperatively to 2 ± 2 mm postoperatively, demonstrating effective correction of radial shortening. Normalization of ulnar variance is essential for preserving distal radioulnar joint stability and preventing ulnar impaction syndrome. The mean postoperative loss of radial deviation was 2.8 ± 2°, with the majority of patients showing minimal loss, consistent with previous studies [13,14].

The distribution-based analysis further supports these findings, with a marked shift of patients from abnormal preoperative ranges to near-normal postoperative values across all parameters. The majority of patients achieved optimal ranges for palmar tilt, radial height, radial inclination, and ulnar variance, indicating effective anatomical restoration with volar plating. This shift toward near-normal anatomical parameters highlights the reliability of volar LPF in maintaining reduction, even in potentially unstable fracture patterns.

According to Sarmiento’s modification of Lindstrom’s grading, 70% of patients had excellent outcomes and 30% had good outcomes, with no fair or poor results. These findings are comparable to Khan et al. [16], who reported similar proportions of excellent outcomes, although Hanumanthappa et al. [13] reported a higher proportion of good outcomes. Residual deformity was insignificant in 70% of cases and slight in 30%, with no moderate or severe deformity observed. This is better compared to Kotian et al. [14], suggesting improved anatomical restoration in the present study. The high rate of excellent outcomes in this study may be attributed to precise surgical technique, stable fixation, and adherence to radiological parameters during reduction.

Overall, the present study demonstrates that ORIF with volar plating provides effective restoration of radiological parameters and achieves stable fixation with minimal residual deformity. The findings support the effectiveness of this technique in achieving near-anatomical reduction and favorable radiological outcomes. Compared to other modalities such as external fixation and percutaneous pinning, volar locking plates may provide stable fixation and facilitate early mobilization following anatomical reduction. Additionally, early mobilization facilitated by stable fixation helps reduce joint stiffness and promotes faster functional recovery.

Although functional outcomes were not assessed in this study, previous literature suggests a correlation between restoration of radiological parameters and improved clinical function. However, the study has several limitations, including a relatively small sample size, absence of functional outcome assessment, lack of long-term follow-up, and absence of a comparative treatment group. Selection bias may also be present due to the inclusion of surgically managed patients only. Radiological evaluation was limited to the immediate postoperative period; therefore, maintenance of reduction, fracture union, late collapse, implant-related complications, and long-term wrist function could not be assessed. Additionally, surgeries were performed by two independent surgeons, which may have introduced variability in outcomes. Interobserver reliability analysis for radiographic measurements was also not performed. These limitations should be addressed in future larger studies with long-term clinical and functional evaluation.

## Conclusions

The present study demonstrates that DRFs are more commonly observed in older individuals, with a higher incidence in females, likely related to osteoporosis and low-energy trauma such as falls on an outstretched hand. ORIF using a volar locking plate was found to be a safe and effective treatment modality, providing reliable restoration of key radiological parameters, including palmar tilt, radial height, radial inclination, and ulnar variance. Favorable radiological outcomes were observed in the majority of patients, with 70% achieving excellent and 30% achieving good results according to Sarmiento’s criteria. The procedure resulted in near-anatomical reduction with minimal residual deformity, with 70% of patients showing insignificant deformity and 30% showing slight deformity and no cases of moderate or severe deformity. Stable fixation achieved through volar plating facilitates early mobilization and rehabilitation, making it a dependable and effective option in the management of DRFs. Further studies incorporating functional outcomes and long-term follow-up are recommended to validate these findings.
